# Enhancing digital readiness and capability in healthcare: a systematic review of interventions, barriers, and facilitators

**DOI:** 10.1186/s12913-025-12663-3

**Published:** 2025-04-04

**Authors:** Norah Alotaibi, Christine Brown Wilson, Marian Traynor

**Affiliations:** 1 Department of Nursing Administration and Education, Collage of Nursing, Shaqra University, Shaqra, Kingdom of Saudi Arabia; 2 School of Nursing & Midwifery, Queen’S University Belfast, Belfast, UK; 3 Faculty of Medicine Health & Life Sciences, Queen’S University Belfast, Belfast, UK

**Keywords:** Digital readiness, Digital capability, Health professionals, Technology

## Abstract

**Introduction:**

The rapid integration of digital technologies in healthcare requires healthcare professionals to be digitally ready and capable. This systematic review aims to identify interventions that improve digital readiness and capability among health professionals and to understand the barriers and facilitators they encounter during this digital transformation.

**Methodology:**

A mixed-methods systematic review was conducted following the Joanna Briggs Institute (JBI) guidelines. We searched five databases CINAHL Plus, MEDLINE, EMBASE, PsychINFO, and Web of Science. The review used the Unified Theory of Acceptance and Use of Technology (UTAUT) framework to investigate factors influencing technology adoption. Studies were selected based on predefined inclusion and exclusion criteria, focusing on health professionals' digital capability in healthcare settings. Quality assessment was performed using the MMAT checklist, and data were analysed and synthesized to extract relevant themes and sub-themes.

**Results:**

Initially, 1140 studies were identified, with 21 meeting the inclusion criteria after screening. These studies, published between 2017 and 2023.The results were categorized into four main themes: Performance Expectancy, Effort Expectancy, Facilitating Conditions, and Social Influence, with two sub-themes. The studies indicated that technology positively impacts job performance, facilitating acceptance among healthcare professionals. Ease of use was crucial for technology acceptance, while complexity and multiple logins were significant barriers. The importance of sufficient training and organizational support was highlighted to enhance digital competency and address technical issues, with inadequate training and infrastructure being major barriers. Social influence, including motivation of healthcare workers and shared decision-making, played a significant role in technology acceptance.

**Conclusion:**

This review highlights critical factors influencing the digital readiness and capability of healthcare professionals. Interventions enhancing performance expectancy, addressing effort expectancy, improving facilitating conditions, and leveraging social influence are essential for successful digital health adoption. Future research should develop comprehensive frameworks to overcome barriers and promote digital health readiness. Integrating specialized training into educational programs is crucial for preparing healthcare professionals to navigate the evolving digital landscape.

**Supplementary Information:**

The online version contains supplementary material available at 10.1186/s12913-025-12663-3.

## Background

Digital health encompasses the use of information and communication technologies to improve human health and healthcare delivery [1]. The acceleration of the development of digital solutions and their integration into healthcare has become a crucial aspect of the work of health professionals requiring them to adapt to new digital technologies [2, 3]. Healthcare professionals across the globe are therefore navigating a rapidly evolving landscape, where new digital interventions are continuously being integrated into the routine work of healthcare [4]. This presents a challenge as the need to acquire new capabilities and skills in order to effectively utilize digital technologies and provide the best possible care to their patients is managed [5]. This includes being able to navigate and utilize electronic health records, telemedicine platforms, wearable devices, and other digital tools [6]. These technologies promise to improve accessibility, efficiency, and the overall quality of healthcare services, revolutionizing the way health professionals interact with and treat patients.

The World Health Organization has accelerated its plans towards establishing a global strategy on digital health 2020–2025 [7]. The WHO digital health strategy is built on a concept of improved health for everyone, everywhere by supporting the development and adoption of person-centered digital health solutions that are appropriate, affordable, accessible, scalable, and sustainable. It is also creating infrastructure and applications that allow countries to use health data to promote health and well-being and to achieve the health-related Sustainable Development Goals [7]. Among the countries that have set a precedent to adapt digital health in their healthcare system health are Australia [8], England [9] and Saudi Arabia [10].

The integration of digital health solutions into the healthcare system has become increasingly important as it offers numerous benefits in improving accessibility, efficiency, and quality of healthcare services [11]. However, the incorporation of these systems does not come without its challenges. Integration of digital capability in health professionals’ workplaces is dependent on user proficiency and competence [5]. Health care professionals must have the necessary proficiency and competence to effectively utilize digital technology in their workplace [12]. They need to be digitally proficient in order to leverage the benefits of these technologies and improve patient outcomes [3]. Moreover, health care professionals have expressed concerns related to the use of technology at the point of care, highlighting the need for investment in implementation and addressing these concerns [13]. By involving healthcare professionals as end-users in the development of digital systems, healthcare organizations can ensure that these systems are fit for purpose and meet the needs of healthcare professionals [14]. Investing in professional development opportunities for healthcare professionals to develop digital capability is also essential. This will empower healthcare providers to effectively use digital technology in their work and contribute to the advancement of healthcare [6].

Digital capabilities in the context of health professionals are defined as the proficiency and competence required to effectively use technology to improve care delivery and outcomes [12]. This digital acumen among healthcare professionals is vital for the successful integration of digital tools, which in turn can lead to superior patient care and the enrichment of healthcare quality [12]. However, little is known about measures and interventions that enhance the readiness and capability of healthcare professionals, as well as the barriers and facilitators they encounter. Therefore, by identifying these factors and highlighting effective interventions, the way may be paved for a smoother transition into a digitally enabled healthcare future, ensuring that health care professional capability is promoted, and high-quality care is provided [15].

### Question and objectives

This systematic review aims to examine strategies and interventions that support digital readiness and capability among healthcare professionals. It also seeks to discern and shed light on the obstacles and enablers encountered by these professionals as they embrace the digital health paradigm. Therefore, this review asks the question:

- **What are the critical components of digital readiness in health professions, and in what ways do identified barriers and facilitators affect digital capability?**

The objectives of this review are to:

1. **identify what constitutes digital readiness in health professions.**
2. **examine the barriers and facilitators that promote digital readiness in health professions.**

## Methods

This mixed-methods systematic review was performed according to the guidelines of the Joanna Briggs Institute (JBI) Manual for Evidence Synthesis. By utilizing JBI’s guides, researchers can systematically analyze various aspects of the papers, such as study design, participant selection, data collection methods [16]. The protocol was registered with PROSPERO (CRD42023461309).

Five medical and academic databases were used in this review: Cumulative Index to Nursing and Allied Health Literature (CINAHL Plus), Medical Literature Analysis and Retrieval System Online (MEDLINE), Excerpta Medica dataBASE (EMBASE), Psychological Information Database (PsychINFO), and Web of Science. These five databases were selected following critical discussion with the supervision team and a subject expert librarian to ensure that they were the most appropriate to extract relevant studies on digital health. The search involved manually reviewing the retrieved studies’ reference lists, reading the full text to ensure no relevant studies were missed [17, 18]. Additionally, Boolean operators (OR & AND) were used to link the search keywords, and the wildcard symbol “*” was employed to expand the search [19]. During the development of the search strategy an experienced health information librarian was consulted [20].

**Fig. 1:**
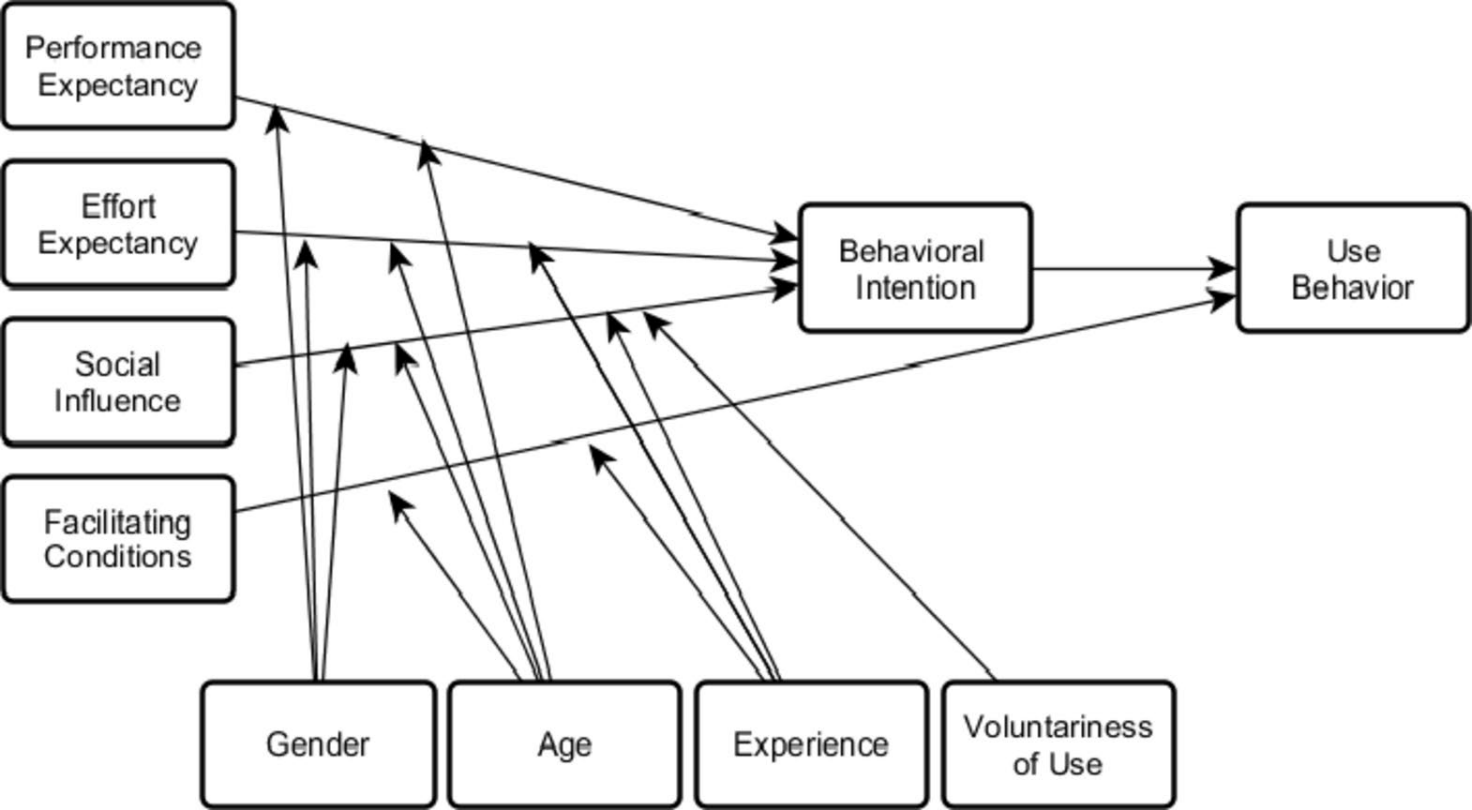
Unified Theory of Acceptance and Use of Technology (UTAUT). Source: [21]

The systematic review used the Unified Theory of Acceptance and Use of Technology (UTAUT) framework (Fig. 1) to investigate behavioral intention with respect to the use of technology and explaining how and why healthcare professionals adopt technologies. The theory was developed by reviewing and integrating eight dominant theories and models, namely: the Theory of Reasoned Action (TRA), the Technology Acceptance Model (TAM), the Motivational Model, the Theory of Planned Behaviour (TPB), a combined TBP/TAM, the Model of PC Utilization, Innovation Diffusion Theory (IDT), and Social Cognitive Theory (SCT). These contributing theories and models have been widely and successfully used by many previous studies of technology or innovation adoption and diffusion across various disciplines, including information systems, marketing, social psychology, and management. Venkatesh et al. [21] presented results from a six-month study of four organizations in their original article, which showed that the eight contributing models explained between 17 and 53% of variance in user intentions to use IT. However, UTAUT was found to outperform the eight individual models with an adjusted R2 of 69 per cent [21].

The adoption and use of technology have been extensively studied in recent years, leading to the development of various analysis models [22]. The Unified Theory of Acceptance and Use of Technology model has emerged as one of the most comprehensive and widely used models in this field [23]. This model integrates constructs from other theories and models to provide a comprehensive framework for understanding technology adoption and acceptance. Additionally, the UTAUT model has been compared with other former theories and models to evaluate its effectiveness and applicability in different contexts. Furthermore, the UTAUT model has been extended and modified to address specific gaps and issues when applied to various contexts [22]. Furthermore, the UTAUT model has been extended and modified to address specific gaps and issues when applied to various contexts [22].

According to the UTAUT model (shown in Fig. 1), there are four main factors that directly affect a person’s intention to use a particular system. These factors are performance expectancy, effort expectancy, social influence, and facilitating conditions. Additionally, the model suggests that these factors can be influenced by gender, age, experience, and voluntariness of use. By analyzing the presence of each of these factors in a real-world environment, researchers and practitioners can understand an individual’s intention to use a specific system and identify the key factors that influence acceptance in any given context [21]. Table 1 summarizes the definition of the core constructs of UTAUT [24].

**Table 1:** The core constructs of UTAUT as defined by Chang [24]

Constructs	Definition
Performance expectancy	The degree to which an individual believes that using the system will help him or her to attain gains in a job
Effort Expectancy	The degree of ease associated with the use of the system.
Social Influence	The degree to which an individual feels that it is important for others to believe he or she should use the new system.
Facilitating Conditions	The degree to which an individual believes that organizational and technical infrastructure exists to support the use of the system.

The Unified Theory of Acceptance and Use of Technology (UTAUT) offers several advantages in healthcare settings, particularly in enhancing the adoption of various health technologies. By systematically analysing the factors that influence technology acceptance, UTAUT provides a structured approach for stakeholders to improve healthcare delivery and patient outcomes. Specifically, the framework identifies key predictors of technology adoption. Performance Expectancy (PE) is a significant determinant of usage intention as it reflects the perceived enhancement in performance. Effort Expectancy (EE) emphasizes that the ease of use of a technology can significantly drive its adoption. Social Influence (SI) highlights the role of peers and organizational culture in encouraging acceptance among healthcare professionals [25–27]. 

Additionally, UTAUT’s flexibility allows it to be adapted across various healthcare contexts, such as telemedicine and e-health systems, ensuring its relevance to different settings and populations [26, 28] The framework also provides actionable insights for policymakers and healthcare providers to design targeted interventions that address specific barriers to technology adoption, ultimately enhancing patient care and operational efficiency [25, 29]. Although existing literature has offered limited comparisons between UTAUT and other models like the Technology Acceptance Model (TAM) and Innovation Diffusion Theory (IDT), recent study have begun to explore these relationships more comprehensively [30]. Both TAM and UTAUT emphasize the importance of perceived usefulness and ease of use as determinants of technology adoption, yet UTAUT extends these concepts by incorporating constructs such as social influence and facilitating conditions, which are particularly critical in digital transformation contexts [31]. In contrast, while IDT primarily focuses on the characteristics of innovations and their diffusion, UTAUT offers a broader perspective by considering user acceptance and usage behaviour an essential distinction for understanding technology adoption in diverse environments [30]. TAM primarily emphasizes perceived usefulness and ease of use [32], UTAUT incorporates social influence and facilitating conditions, which are particularly relevant in healthcare settings where organizational support and infrastructure play a crucial role in digital adoption. Additionally, IDT focuses on diffusion across populations rather than individual user adoption, making UTAUT a more suitable framework for our study’s objectives.

The holistic approach embedded in UTAUT therefore allows for a more nuanced understanding of user behaviour, making it applicable across diverse contexts, including e-learning and healthcare technologies [25, 31]. UTAUT has been extensively validated through systematic reviews and meta-analyses, demonstrating its reliability in predicting technology adoption outcomes [25, 33]. For instance, a meta-analysis revealed Performance Expectancy as the most significant predictor of Usage Intention in healthcare settings [25]. Moreover, the model has been adapted to various fields, such as Islamic financing and higher education, showcasing its flexibility in addressing specific user needs and cultural contexts [34, 35]. This adaptability enhances its relevance in rapidly evolving technological landscapes.

Furthermore the theoretical framework is grounded in the Unified Theory of Acceptance and Use of Technology (UTAUT) and this framework was therefore chosen in this review for its comprehensive approach to understanding technology adoption, particularly in digital health contexts [21]. UTAUT considers critical factors such as social influence and facilitating conditions, which are highly relevant to the healthcare sector, where adoption decisions are shaped by organizational, cultural, and social dynamics [24]. UTAUT also provides valuable insights into the motivations and factors influencing individuals’ adoption of technology, especially those that contribute to accelerating this process [24]. These features make UTAUT a suitable and robust framework for analysing digital readiness among healthcare professionals and aligned with the objectives of this review.

### Inclusion and exclusion criteria

Digital capability refers to the ability of health professionals to effectively and confidently use digital technologies in their practice [36]. Inclusion and exclusion criteria were developed using the PEO (Population, Exposure, Outcome) framework (Table 2). This study included health professionals as the target population and studies focusing on digital capability, digital competence, or digital readiness in healthcare settings. Studies were excluded that focused on health professions students or patients and studies that focused on digital capability in non-healthcare professions.

**Table 2:** Inclusion criteria and exclusion criteria

Include	Exclude
**Population**	
• Health professionals	• Health professions students. • Patient.
**Exposure**	
• Digital capability. • Digital readiness • Digital health knowledge • Digital capability frameworks • Digital health • Digital Literacy*	• Information literacy • Health literacy • E-learning • Simulated learning • Technology-enhanced learning • Peer support programmed • Development and/ or testing of scales
**Context**	
• Only primary studies published in English language. • Studies that focus on digital readiness and or digital literacy and or digital capability. • Studies reporting barriers and facilitators of using technology in practice. • Studies exploring use of technology or digital devices in practice.	• Non-English studies • Social media • Patient outcomes • Abstract only papers • Books
**Outcome**	
• Recommendations or interventions that contribute to enhancing digital capability and increasing digital readiness in health professions. • Barriers and\ or facilities that promote digital readiness in health professions.	
**Time frame**: No restrictions	

*= MeSH = Medical Subject Headings

The inclusion criteria were designed to ensure that only studies directly relevant to digital readiness and capability in healthcare settings were selected. This means that we included studies focusing on health professionals and topics such as digital health knowledge, digital capability frameworks, digital health, and digital literacy. These criteria ensure that the research directly addresses our objective of understanding the factors that enhance digital readiness among healthcare professionals.

Conversely, our exclusion criteria were established to filter out studies that do not align with our research focus. For example, studies that concentrate on information literacy, health literacy, e-learning, simulated learning, technology-enhanced learning, peer support programs, or the development and testing of scales were excluded. Although these topics are valuable in their own right, they do not specifically pertain to the digital readiness and capacity of healthcare professionals, which is the central focus of our review.

By applying these criteria, we aimed to capture the most relevant evidence that directly informs our understanding of digital readiness in healthcare, ensuring both the clarity and relevance of our findings.

The following Keywords were developed by authors and a subject specialist librarian (Table 3). Five databases were searched: CINAHL, MEDLINE, EMBASE, PSYCHINFO and the Web of Science. Selection of these databases is strategic due to their comprehensive coverage of healthcare and medical literature, including peer-reviewed and authoritative sources that are critical for health studies [37]. These databases provide multidisciplinary insights, essential for a holistic understanding of the field, and advanced search capabilities to efficiently identify relevant studies. Furthermore, they offer a mix of historical and current research, enhancing the depth and breadth of the review, with the Web of Science providing an added advantage of international coverage, thereby offering a broad spectrum of global perspectives [38]. CINAHL specifically includes nursing and allied health literature, directly relevant to health care professionals (Canaling). This careful selection ensures the research is built upon reliable, diverse, and relevant academic scholarship [37].

**Table 3:** Search terms

Search terms				
Keywords				
1-“digital readiness” OR “Digital capability” OR “digital capability frameworks” OR “Digital health knowledge” OR “Digital health (MH)” OR “Digital Literac*” OR “Digital capabilit*” OR “Digital skills” OR “Digital learning” OR “Data Literac*” OR “Digital collaboration” OR “Digital environment” OR “Digital ethics” OR “Digital competenc*” OR “e-literacy” OR “Technology enhanced learning” OR “Digital literacy project” OR “Digital infrastructure” OR “ICT proficienc*” OR “ICT productivity” OR “information literacy (MH)” OR “Media literac*” OR “Digital research and problem solving” OR “Digital innovation” OR “Digital participation” OR “ Digital teaching” OR “Digital learning and development” OR “Digital identity management” OR “Digital identity” OR “Digital wellbeing” OR “Digital self-actualis*” OR “Digital best practise” OR “Digital health care currcul*”	AND	2- “health professional*” OR “nurse*” OR “health care provider*” OR “care giver”	AND	3- “barrier*” OR “facilitator*” OR “measure*” OR “factor*” OR “Perception(MH)” OR “experience*” OR “inhibitors” OR “Influencing”

(* = MeSH = Medical Subject Headings)

### Selection of studies

**Fig. 2:**
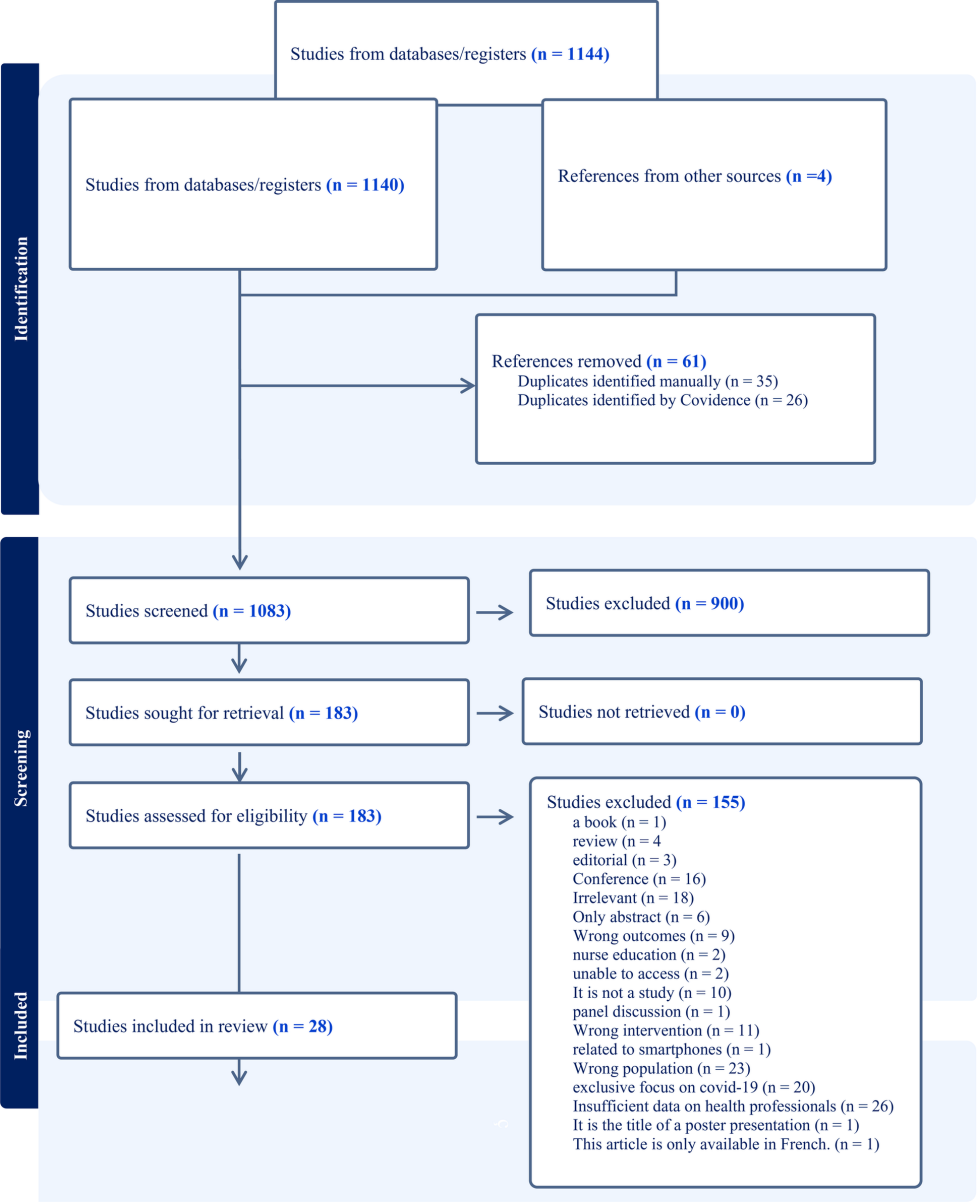
The PRISMA of studies as shown in Covidence

The studies were identified and evaluated according to the preferred reporting items for systematic reviews, (PRISMA) 2020, to ensure best practice in data extraction, analysis, and reporting [39]. After conducting the search all the relevant studies were imported to Endnote software (version 20) which is a reference manager program. By using Endnote duplicated studies were identified and removed. The researcher then manually screened for duplications. Finally, to ensure accuracy and manage the large number of studies the researcher exported studies into Covidence [40]. Thereafter, the results were reviewed in three phases. In phase one, two people screened the title/ abstracts independently against the inclusion/ exclusion criteria, with conflict resolved by a third reviewer. Phase two, two people read full text of the remaining studies independently rejecting studies with reasons. A PRISMA flow diagram summarises the selection process (Fig. 2).

### Quality assessment

Eligible studies were assessed for quality using the Mixed Methods Appraisal Tool (MMAT, version 2018). This was employed to principally evaluate quality mixed methods studies and also includes an evaluation of qualitative and quantitative methods [41]. Although there are several quality assessment tools that can be used to critically appraise studies, the Tool (MMAT) is an essential resource that enables critical appraisal of mixed methods research in nursing. It strengthens methodological rigor and aids in planning and conducting future mixed studies [41]. In this systematic review, the MMAT, for mixed methods, qualitative and quantitative randomized control trials, quantitative non-randomized and quantitative descriptive were used. The MMAT includes two screening questions for every study. followed by a category dictated by the study design; for each design five questions. Each of (MMAT) Questions were rated as ‘no’, ‘yes’, and’ can’t tell’ [42]. The risk of bias for each study was determined based on the percentage of questions answered yes. According to Hong et al. scores will range from 0% (0/7 yes) to 100% (7/7 yes).

### Data extraction

Data extraction forms were developed based on the objectives of this systematic review to collect data from the included studies, including study characteristics and outcomes [43]. The extracted data included data from each study and also the following information: studies characteristic: study title, authors names, years of publish, study design, country, sitting, and sample size, geographical location, key findings and study limitations (Table 4, Appendix 1).

The key findings from each study were extracted and organized into a comprehensive table, categorically divided into the four core constructs of the UTAUT framework: Performance Expectancy, Effort Expectancy, Facilitating Conditions, and Social Influence. Notably, the analysis also considered variables that might be influenced by factors such as gender, age, experience, and voluntariness of use.

### Data analysis

The Unified Theory of Acceptance and Use of Technology is a theoretical framework that aims to explain individuals’ acceptance and use of technology [43]. It was developed to address the limitations and gaps in previous models and theories related to technology adoption [22].

The studies included in this review were analysed, using (UTAUT). First the studies were read in depth and data extracted such as demographic details, methodological details, limitations, and significance of relationships between the constructs. The findings of the study were then compared with the core determinants in the UTAUT model. Finally, a determination was made as to the extent to which the UTAUT model explained technology adoption and acceptance in the study.

## Results

A total of 1140 studies were identified in the initial database search. Four additional studies were incorporated based on references from the existing studies, and 61 duplicate studies were subsequently removed. Utilizing the PRISMA procedure, the remaining 1083 studies were screened based on title and abstract, leading to the exclusion of 900 studies. Subsequently, the eligibility of the remaining 183 studies was assessed in full text, resulting in the inclusion of 28 studies in the review and the exclusion of 155 studies.

The 28studies used five different study designs, which all went through a quality assessment. Seventeen of the included studies were classed as having a high quality according to the MMAT checklist [44–60].

Four of the included studies was classed as having a moderate quality [61–64], Furthermore, seven of the included studies were classed as having a low quality [65–71].

After a critical discussion with the two academic authors of this review, studies with a low quality were excluded in an attempt to use only information from high and moderate quality studies (See Appendix 2 for the full summary of MMAT Critical Appraisal Tool).

### Characteristics of included studies

In this systematic review a total of 21 studies were identified among which 12 were qualitative [46, 48–53, 58–60, 62, 63] and 4 quantitative [44, 56, 57, 64], and 5 mixed and multimethod studies [45, 47, 54, 55, 61] (Appendix 1).

These studies were published between 2017 and 2023. Three studies were from Canada, followed by two from Australia and also included studies from Finland, Nigeria, England, Taiwan and China, Cape Town, South Africa, German, Polish and Greek. The results are presented based on the four core constructs of UTAUT to discuss outcomes related to users’ behavioural intention to use the technology. Which is Performance Expectancy, Effort Expectancy, Facilitating Conditions and Social Influence. Therefore, the number of themes is four and 2 sub-themes were extracted.

#### Theme 1. Performance expectancy

Performance expectancy is a key construct in the Unified Theory of Acceptance and Use of Technology, which aims to explain individuals’ intentions to use technology in organizational settings [23]. Performance expectancy refers to the extent to which individuals believe that using a particular technology will help them perform their tasks more effectively and efficiently [23].

In this review, insights from seven studies [44, 47, 50, 57, 58, 61, 64] collectively suggest a noteworthy connection between users’ perceptions of enhanced performance and their inclination to embrace the technology. According to [47] a key driver of acceptability cited by frontline health workers (FHWs)was the perceived usefulness in improving the quality of health care provision.

Chang et al. [44] investigated the factors that affect nurses’ intentions to use information systems for nursing. The results of the study confirmed the importance of performance expectancy in predicting the usage intentions of nursing information systems. The study also found that operational ease is more important to users in accepting societies, for example, China, indicating a need for tailored strategies.

 Singh et al*. *[58] reported that health care professionals wanted to help patients achieve meaningful personal goals. However, health care professionals varied in how they pursued this aim, which influenced how they engaged with the digital tools. For example, according to Singh et al. some health care professionals had a high or low behavioral intention and, for some, it changed over time. In the same way [64] found that both ability and motivation play had an important and positive role in the adoption and behavioral intention of digital health care by influencing performance expectancy. Health care professionals that perceived the digital health tools to be useful and relevant to their job had a significantly more positive attitude formation toward and intention to use the system. However [64], also found that familiarity with online experience did not play a role in the perceptions and adoption digital health tools. Conversely [57] reported that the majority of health professionals had a positive attitude as a result of their experience, including high digital literacy, which played a positive role in the acceptance of digital tools.

 Burridge et al. [61] findings demonstrate that nurses face daily challenges with electronic medical records (eMRs) that integrate paper-based activities for efficient and timely care. Despite the push for a paperless environment, nurses realize the value of electronic systems considered as complementary methods with paper systems instead of opposing [61]. Similarly [50], found that participants also valued the shared electronic documentation to inform and facilitate patient care, enabling more efficient work by reducing the time needed to complete documentation and through preventing mistakes and loss of patient data.

#### Theme 2. Effort expectancy

Effort expectancy is a crucial factor in the Unified Theory of Acceptance and Use of Technology. Effort expectancy refers to the perceived ease of use and the perceived effort required to use a specific technology [21]. It measures the user’s perception of how complex or difficult it would be to use a technology. Effort Expectancy plays a key role in determining an individual’s intention to use and accept technology [21].

Three studies in this review [44, 49, 63] clarify that the degree of ease associated with the use the digital tools is an important factor in technology acceptance. Moreover [63] also found that effort expectancy lack of emphasis on usability, the complexity of use emerged as a significant barrier to acceptance contributed to unclear use digital tools.

 Lloyd et al. [49], reported that the need for multiple logins and sign-outs, challenged users and led to a loss of productivity through wasted time and is exemplified in the quotes. ‘We still have different logins for different providers that means we waste time logging in etc.’ (Medical #483); ‘If you are not using the program, it shuts down so [you] have to repeatedly sign in throughout the day. While sitting at desk where I am using the program it will shut down. IT [Information Technology] department will not extend the time it is open. This makes the program very difficult and user unfriendly’ (Nurse #150); and ‘Loss of productivity. Takes 15 min to log in to all the necessary electronic systems and I can out-type the computer every time so need to slow down my typing to avoid errors’ (Medical #528). Moreover [44], study found that information literacy has a positive impact on effort expectancies, promoting the belief that using nursing information systems can enhance performance and increase usage intentions.

#### Theme 3. Social influence

Social influence is one of the key constructs in the UTAUT model that refers to the degree to which an individual feels that it is important for others to believe they should use the new system. Two of the included studies mentioned to social influence [59, 60]. The main influence factors mentioned that convince the individual can encourage others to follow suit in healthcare were motivating healthcare workers and patients’ healthcare providers by clarifying the added value of using a digital care platform, clear business case with vision, demonstrating effectiveness, using an implementation guide, and educating patients and health care providers about how to use digital health tools [59]. In addition, Shared decision-making and patient-cantered care were play an important role in establishing perceived value that motivates the team to embrace technology [60].

#### Theme 4. Facilitating conditions

In the Unified Theory of Acceptance and Use of Technology, facilitating conditions refer to the degree to which individuals perceive that they have the necessary organizational and technical infrastructure to use a specific technology. Facilitating conditions play an important role in the UTAUT model as they influence the intention to use a technology [72]. Facilitating conditions are important in the UTAUT model because they determine whether individuals have the necessary resources and support to effectively use a technology [72].

### Insufficient training

Insufficient training can act as a significant barrier to the adoption and acceptance of technology as was evident in several studies: [45, 51, 52, 54–56, 60, 62].

 Shiferaw et al. [56] reported that insufficient training in problem-solving in digital health issues, lead to low basic digital competency among health care professionals, particularly in routine problem-solving, safety, and communication. The majority lacked basic technical skills for hardware and software issues. Also, sex, educational status, profession type, and years of experience were significant factors which impacted behavioral Intention. Males were 3.9 times more likely to have higher digital competency and education level and positively correlated with digital competency also, younger professionals may be more receptive to changes in the working environment.

Participants in the study by [62] had limited exposure in professional training to digital health during their education. In addition, readiness challenges that related to infrastructure such as digital skill gaps and infrastructure deficiencies led to negative behavioural intention which resulted in poor penetration of digital health by [52].

According to [45] many care professionals had available resources and support for training on virtual care. A few did not initially have infrastructure available to them. A few statements also revealed that those who had previous experience with telemedicine use found the transition to be smooth. In the same context [51] found that the majority of nurses need training to manage digital tools and identification of personnel responsible for managing symptoms; that is facilitating conditions that empower nurses.

Jensen et al. [54] factors influencing lower uptake were computer unavailability, staff allocation, low literacy, training time, and workload concerns.

Insufficient training and support can hinder the scale-up of digital tools, leading to negative usage outcomes and affecting their intended benefits [60]. Similarly, a study [52] found that inadequate training and lack of on-the-job support contributed to stress and feelings of incompetence, which can lead to resistance to information technology (IT) and technology adoption, both personally and professionally. These conditions may cause individuals to be late technology adopters.

### Organizational and technical infrastructure

Five studies in this review clarify the importance of organizational and technical infrastructure to support the use of the digital system: [48, 53, 57, 60, 63].

 Faujdar et al. [53] illustrates the issues faced by Health care workers (HCWs) during the introduction and maintenance of the eHealth system. They reported a number of technical issues, such as breakdowns of hardware, bugs in the software, and an erratic electricity supply which in turn effects user behaviors. Faujdar et al. [53] reported that HCWs found that the eHealth system did not provide flexibility [48]. emphasizes the importance of providing resources and opportunities for digital competence sharing to help create friendly and safe digital organizational atmosphere.

Privacy concerns have a significant impact on the acceptance of technology. According to findings, individuals are increasingly concerned about their privacy and the security of their personal information. This concern can lead to hesitation and reluctance in adopting new technologies. For example [59], found that doubts about the privacy and security of data, as well as insufficient digital skills of users, can hinder technology acceptance. A study conducted by [63] found that a considerable proportion of participants had concerns about privacy issues related to technology for example, “can violate my own/my relatives’ privacy,” 62.5% (*n* = 15) of participants agreed or strongly agreed with this statement [63]. This indicates that a considerable proportion of participants had concerns about privacy issues. These concerns included the fear of data privacy violations, which can negatively impact the attitude towards technology acceptance [63]. Furthermore [57], found that a negative perception of security and privacy can lead to a less positive attitude towards technology acceptance. Overall, privacy concerns are a significant factor that influences individuals’ attitudes and intentions towards accepting and adopting new technologies. The level of trust individuals has towards technology can greatly influence their attitudes and intentions to adopt new technology. Finally [60], reported that healthcare workers raised concerns about the accuracy of data captured by DHT due to the lack of validation and calibration.

## Discussion

This systematic review sought to investigate the multifaceted landscape of interventions shaping the digital readiness and capability of health professionals across diverse global healthcare settings. The aim of this review was to identify the interventions that support digital capability and digital readiness in health professions and to identify what are the barriers and facilitators that promote digital readiness in health professions. Based on the findings of this review, it can be concluded that constructs including perceived usefulness, social influence, trust, perceived ease of use and facilitating conditions represent the driver’s influencing intention and behavior in the use of digital health tools. Research findings also indicate that insufficient training, infrastructure deficiencies, and usability issues are significant barriers influencing the usage digital health tools.

The findings of the included studies provide insights into users’ behavioral intention to use health care technology, specifically related to the construct of Performance Expectancy [45, 46, 50, 52, 57, 58, 60, 61, 64]. In the review several studies focused on the construct of Performance Expectancy in the context of health care technology adoption [73]. In a study based on the UTAUT2 model, researchers have explored the factors that influence users’ intention to use digital health information system. Based on their findings, they determined that effort expectancy, social influence, perceived risk, and habit have a significant impact on users’ behavioral intention to use the technology [74]. The exploration of how health professionals pursue meaningful goals through digital tools shows that perceived usefulness is dynamic in nature. It is worth noting that this review found that both ability and motivation, play an important and positive role in the adoption and behavioral intention of digital health care by influencing performance expectancy [64].

The findings from the review consistently demonstrated that users’ perceived benefits and expectations of performance strongly influence their intention to adopt and use health care technology [58, 64]. The results are in line with previous studies that have highlighted the importance of Performance Expectancy in shaping users’ intentions to adopt technology [75]. For example, a review of the literature on health information technology found that 92% of the articles reached positive conclusion that perceived usefulness significantly influences the intention to adopt and use health care technology. Moreover, a study by [76] revealed that when users perceive a technology as beneficial, they are more likely to embrace it and integrate it into their daily practice [76]. Whereas when users perceive low usefulness or benefits from a health care technology, they are less likely to adopt and use it) [77]. Other studies have also supported these findings. For example, in a study by [78] they found that perceived usefulness was a key determinant of technology acceptance and adoption in the healthcare context [78]. Based on the studies included in the review, it is evident that if health care professionals, as users of the technology, believe that technology will enable them to perform more effectively in their role, this will strongly influence their intention to adopt and use it.

In addition to Performance Expectancy, some studies also examined the impact of Effort Expectancy and Facilitating Conditions on users’ behavioral intention to use healthcare technology [79]. However, it is important to note that there was limited research available on these constructs within the context of healthcare technology adoption [80]. The findings of this systematic review clarify that the degree of ease associated with the use the digital tools is an important factor in technology acceptance. The concept of facilitating conditions refers to the external factors or resources that support and enable the effective use of technology. The included studies in this review identified that facilitating conditions such as specialized training and technical support play a significant role in the acceptance and use of digital health systems [56, 62]. Similarly, some studies in the literature reported that the availability of technical support, training programs, and adequate resources positively influenced users’ intention to adopt and use digital health systems [81]. However, another study [82] found that a lack of facilitating conditions, such as limited technical support or inadequate training, hindered the adoption and use of digital health systems which are considered barriers. These complementary findings highlight the importance of providing a supportive environment for users to successfully adopt and use digital health systems. Organizations should invest in providing necessary interventions to normalize facilitating conditions across consumers.

The studies within this review emphasize that digital readiness is a multifaceted construct, with a number of facilitating conditions including technical infrastructure [48, 53]. Similarly, other studies have also found that infrastructure plays a foundational role [6, 83]. Organizational and technical infrastructure appears therefore to be the backbone of digital readiness. Similar to the findings from this review [84, 85], also illustrate the challenges faced due to technical issues and insufficient training, emphasizing the need for a facilitating infrastructure. This theme echoes across various settings, including studies by [51, 86] highlighting the importance of resources and training support. Significantly, organizational policies and readiness challenges, as illustrated by [87, 88] further emphasize that effective interventions necessitate a holistic approach.

This systematic review highlights the need for future research to explore an approach or framework capable of tackling the obstacles and facilitating the enhancement of digital capacity and readiness. Such endeavors are crucial to ensure the efficacy and success of digital interventions. A key component arising from the review emphasizes the critical role of digital health training in enhancing the capacity and readiness of healthcare professionals [46, 51, 52, 54, 56, 60, 62]. The review findings indicate a clear correlation between training interventions and the enhancement of digital competency among healthcare professionals. This underscores the importance of integrating specialized digital health training into educational programs [87, 89]. This incorporation will ensure that future health professionals are adequately equipped to navigate the complexities of a digital healthcare landscape [3].

In addition to the barriers mentioned above the included studies also highlighted several challenges and barriers to the implementation of digital health tools. One common challenge identified was the resistance to change among healthcare professionals [52]. Health care professionals were hesitant to adopt new technologies and preferred traditional methods of healthcare delivery [52]. Additionally, the lack of technical skills and knowledge among healthcare professionals was also identified as a barrier to acceptance of digital health tools [53, 56]. Other barriers included concerns about privacy and security of patient information, as well as issues related to the usability and functionality of the digital health tools [59, 63]. An interesting finding is the role of experience as a variable influencing the perception and acceptance of digital health tools, [57]. Similarly, professionals with higher levels of digital literacy and experience exhibited more positive attitudes toward digital interventions [90]. This highlights the importance of tailoring training programs to accommodate varying experience levels, ensuring that both novice and experienced professionals benefit optimally from digital health education [91].

### Strengths and limitations the review

A notable strength of this review is that it encompasses a diverse range of digital health tools across various healthcare systems from different countries, which contributes to the robustness and generalizability of the findings. Moreover, the review benefits from comprehensive database searches incorporating five high-quality databases, well-regarded for their extensive coverage of medical research literature. This extensive search strategy, combined with the inclusion of studies meeting rigorous quality criteria, enhances the reliability and validity of the results. Importantly, a specific publication timeframe was not imposed, thereby allowing the inclusion of studies from all periods and ensuring a comprehensive capture of relevant evidence.

However, certain limitations should be acknowledged. Due to resource constraints, the search was restricted to papers published in English, which may have led to the exclusion of pertinent literature in other languages. Additionally, the last search in the databases was conducted on April 29, 2023. Another limitation is that the review exclusively applied the Unified Theory of Acceptance and Use of Technology (UTAUT) as the theoretical framework, which, while robust, may not capture all dimensions of digital transformation. Furthermore, the focus was confined to topics specifically related to digital health knowledge, capability frameworks, digital health, and digital literacy; as such, studies examining broader aspects of digital readiness may have been excluded.

Overall, while the methodological approach was rigorous and well-grounded, there are some limitations and therefore any future research may benefit from exploring additional theoretical perspectives and a broader range of related topics.

### Implications for practice and future research

Based on the findings of this systematic review, there are some recommendations that could be considered to address the identified barriers and enhance digital readiness and capability among healthcare professionals. For example, to enhance training and educational programs, dedicated digital health modules, that integrate specialized training, could be incorporated into undergraduate curricula and continuing professional development programs. The recommendation is that these modules are tailored to various levels of digital literacy, ensuring that both novice and experienced healthcare professionals can effectively engage with digital tools [92, 93]. A further recommendation would involve providing hands-on workshops and simulations, such as practical training sessions, workshops, and simulated learning environment, to enable healthcare professionals to gain familiarity with new technologies and build confidence in their digital skills [94, 95].

Strengthening technical and organizational infrastructure is also crucial. A recommendation to upgrade IT systems by investing in robust IT infrastructure to ensure reliable hardware, software, and network connectivity should be considered. Streamlining digital systems to reduce complexities, such as multiple login procedures, and improving interoperability between different platforms are essential steps [96, 97]. In parallel, establishing dedicated technical support teams to assist healthcare professionals with troubleshooting will also help ensure minimal disruption to digital) [98, 99].

Fostering a supportive organizational culture is equally important. Healthcare organizations should promote digital champions by identifying and empowering individuals within teams to lead by example and encourage the adoption of digital tools [100]. Additionally, cultivating an environment that encourages shared decision-making, where feedback from end-users is actively sought and incorporated into digital system enhancements can mitigate resistance to change and foster a collective commitment to digital transformation [101]. Furthermore, organizational leadership plays a crucial role in digital transformation. Implementing digital mentorship programs allows experienced staff to guide their colleagues in learning new technologies, and structured change management ensures that transitions to digital systems are planned and executed smoothly, reducing resistance and confusion [102]. Together, these strategies create a supportive environment that helps everyone adapt to new digital tools.

Addressing privacy and security concerns is another critical area. Organizations should be supported to implement robust security protocols to ensure that digital health systems comply with stringent data protection standards and regularly update these security measures to safeguard patient information and build trust among users [103, 104]. Furthermore, regular training sessions focused on privacy and security best practices are essential to educate healthcare professionals, thereby alleviating concerns and improving confidence in digital systems [105].

Optimizing user experience and system usability is imperative for successful digital adoption. A user-cantered design approach should be employed by engaging healthcare professionals in the design and evaluation of digital tools to ensure that they are intuitive and aligned with clinical workflows [106]. In addition, continuous usability testing should be performed by regularly assessing system interfaces and incorporating user feedback to streamline processes and reduce barriers such as unnecessary complexity and repetitive login procedures [106, 107].

Finally, establishing continuous monitoring and evaluation mechanisms is essential. Implementing systematic feedback loops and performance metrics will enable the continuous assessment of the impact of digital interventions [108]. The data collected through these mechanisms should guide iterative system improvements, ensuring that digital health systems remain effective, user-friendly, and responsive to evolving needs over time [109].

By implementing these recommendations, healthcare organizations can effectively overcome current challenges, promote a culture of digital innovation, and ultimately enhance the quality and efficiency of patient care. Future research should focus on evaluating the real-world impact of these interventions to further refine best practices in digital health readiness and digital capability.

In summary the findings of this systematic review are important for health care organizations and administrators to focus on assessing the readiness of health professionals for digital empowerment and promoting the perceived benefits and potential positive outcomes of technology use among users. The knowledge gained from this review can be applied by countries that face similar challenges in developing the skills of their healthcare workforce for the digital future. For example, the challenges highlighted, such as the shortage of technical skills and training opportunities for health care providers registrants, are likely to be relevant to healthcare industries worldwide [110]. Numerous nations are facing comparable issues as they strive to incorporate digital technologies into their healthcare systems [110]. By recognizing these challenges and the impact on nurse leaders, other countries can gain valuable insights while navigating their own transitions towards digital healthcare [111].

## Supplementary Information

Supplementary Material 1.

Supplementary Material 2.

## Data Availability

No datasets were generated or analysed during the current study.
