# Supplementary material for: Enhancing digital readiness and capability in healthcare: a systematic review of interventions, barriers, and facilitators
Source: BMC Health Serv Res. 2025 Apr 4;25:500. doi: 10.1186/s12913-025-12663-3 (PMC11969766; doi:10.1186/s12913-025-12663-3)
Supplement: Supplementary file 1 — Supplementary Material 1. [file 12913_2025_12663_MOESM1_ESM.docx]

**(Appendix 1)**

**characteristics of the included studies.**

**Table 4.** characteristics of the included studies.

| Title | Author, year and country | Study aims | Study design | Sample size،  health professional classification  center | **Key findings** | **limitations** |
| --- | --- | --- | --- | --- | --- | --- |
| Exploring the perspectives of primary care providers on use of the electronic Patient Reported Outcomes tool to support goal-oriented care: a qualitative study | Singh et al., (2021)  Canada | 1. his study explores enablers and barriers experienced by primary care providers when adopting new digital  health technologies.  ) using the example of the electronic Patient Reported Outcome (ePRO) tool; a mobile application and web portal designed to support goal-oriented care. To better understand implementation drivers and barriers primary care providers' usage behaviours are compared to their perspectives on ePRO utility and fit to support care for patients with complex care needs (. | qualitative study | thirteen providers  from three primary care sites | Three core themes were identified: (1) Perceived usefulness: perceptions of the tool’s alignment with providers’ typical approach to care, impact and value and fit with existing workflows influenced providers’ intention to use the tool and usage behaviour; (2) Behavioural intention: providers had a high or low behavioural intention, and for some, it changed over time; and (3) Improving usage behaviour: enabling external factors and enhancing the tool’s perceived ease of use may improve usage behaviour.  . | There were various challenges faced during the trial initiation, including recruitment delays. The tool used faced technical glitches which negatively impacted the engagement of providers. |
| Stakeholders’ Perceptions of the Implementation  of a Patient-Centric Digital Health Application for  Primary Healthcare in India | Faujdar, et al., (2021)  Chandigarh, India | Health systems are shifting from traditional methods of healthcare delivery to delivery using digital applications.  This change was introduced at a primary care center in Chandigarh, India that served a marginalized population. After  establishing the digital health system, we explored stakeholders’ perceptions regarding its implementation. | qualitative study-  focus group discussions and in-depth interviews. | 20 adults +  6 health workers  HCWs  In a primary  care center | present study illustrates physicians’ perceptions for eHealth as system that less prone to errors and initially tedious, but later useful, supports decision-making and is more reliable, ease follow-up & tracking, ensures basic health care requirements, required infrastructural and technological support, insufficiently comprehensive or automated and stress levels among employees are easier to monitor. While perceptions of  nurse midwives about eHealth system represented in the most useful features which is easy search and report generation | N/A |
| A comprehensive eHealth implementation  guide constructed on a qualitative case study  on barriers and facilitators of the digital care  platform CMyLife | Verweij et al., (2022)  Netherlands | To explore barriers to and facilitators for nationwide implementation and consolidation of CMyLife, a multicomponent,  patient-centred, digital care platform, and to construct a comprehensive implementation guide for  launching digital care platforms in daily clinical practice. | qualitative case study | In this study, it was conducted 23 interviews five were focus group. | - 45 barriers and 41 facilitators were identified. Main barriers were lack of connectivity between information technology systems, changing role for both health care providers and patients, insufficient time and resources, doubts about privacy and security of data, and insufficient digital skills of users. - Main facilitators mentioned were motivating patients and health care providers by clarifying the added value of use of a digital care platform, clear business case with vision, demonstrating effectiveness, using an implementation guide, and educating patients and health care providers about how to use CMyLife. | - This study has limitation, the sample size is small. |
| Attitudes Toward Health Care Virtual Communities of Practice: Survey Among Health Care Workers | Yada et al., (2019)  Canada | The aim is to explore how motivation and ability affect attitudes toward using VCoPs for those working in health care. | Quantitative research | 86 health care workers  At  two Canadian health care conferences. | Using the ELM framework, this research found that both ability and motivation play an important and positive role in the adoption of health care VCoPs. Health care workers that perceived the VCoP to be useful and relevant to their job had a significantly more positive attitude formation toward and intention to use the system.  Familiarity with online social networks and EMRs did not play a role in the perceptions and adoption of a VCoP; experience with CoPs and VCoPs both had statistically significant negative correlations with attitude change.  This means that the higher the experience level, the smaller the change in attitude after experiencing our experimental VCoP.  The lower the experience level with CoPs and VCoPs, the larger the change in attitude | The study' surveys were distributed at conferences, which could have resulted in sample bias towards individuals already involved in healthcare quality improvement. |
| Telemedicine: the experience of health professionals in the supplementary sector | Araújo et al.,  (2023)  Brazil | To know the experience of health care professionals about telemedicine in a supplementary health service. | a qualitative study carried | This study's population comprises the outpatient clinic with 35 professionals: 9 administrative assistants and 26 health professionals, including four family nurses, nine family and community physicians, two dietitians, two psychologists, and nine nursing technicians. . | Participants had limited exposure in Professional Training to digital health during their education. | The study's limitations result from its conduction during a pandemic, providing a specific moment's snapshot |
| Readiness for Delivering Digital Health at Scale: Lessons From  a Longitudinal Qualitative Evaluation of a National Digital Health  Innovation Program in the United Kingdom | Lennon et al.,  (2017)  UK | The aim of our study was to examine barriers and facilitators to implementation of digital health at scale through  the evaluation of a £37m national digital health program: Delivering Assisted Living Lifestyles at Scale” (dallas) from 2012-2015. | Longitudinal qualitative and survey data | The methods included interviews  (n=125) included health care professionals; and social service  managers; staff and volunteers from third sector organizations. | **Key Findings:**  **1-Ubiquitous Readiness Issues:**   - Readiness challenges identified across macro, meso, and micro levels and various sectors. - Not insurmountable but require acknowledgment and addressing for successful large-scale deployment.   **2- Study Strengths and Limitations:**   - Rigorous examination of a national digital health program in the UK. - Grounded in sociological theory (NPT), providing insights into potential barriers. | N/A |
| Attitude towards tele rehabilitation-based therapy services and its associated factors among health professional working in specialized teaching hospitals in Amhara region, Northwest Ethiopia | Sidelil et al., (2021)  Ethiopia | The significance of this study is to assess the attitudes and associated factors of health  professionals about TR services. | An institutional-based cross-sectional quantitative study design | 408 participants from health professionals | **Key Findings:**   - Majority of health professionals (69.9%) had a positive attitude. - Good knowledge of tele-rehabilitation associated with a positive attitude. - Ownership of smartphones significantly associated with positive attitudes. - High digital literacy linked to a positive attitude. - Negative perception of security and privacy associated with a less positive attitude. | N/A |
| Exploring the barriers and facilitators for the use of  digital health technologies for the management of  COPD: a qualitative study of clinician perceptions | Slevin et al., (2019)  Ireland | this study aims to qualitatively explore the barriers and facilitators HCPs perceive for  the use of DHT in the management of COPD. | A semi-structured interview | Participants (n . 32)in  two university hospitals and several general  practitioner clinics.  eight general  practitioners (GPs); four respiratory specialist nurses; four respiratory  Physiotherapists; eight non-consultant hospital doctors  and eight respiratory consultant physicians. | **Barriers to Adoption of Digital Health Technologies (DHT) for COPD Management:**  **1-Data Quality Concerns:**   - HCPs raised concerns about the accuracy of data captured by DHT due to the lack of validation and calibration. - Reliability issues with patient-generated data compared to clinically captured information.   **2- Lack of Evidence-Based Care:**   - Participants expressed reluctance to recommend DHT due to a lack of evidence demonstrating significantly improved outcomes. - Absence of clinical guidelines contributes to hesitation in prescribing DHT for COPD management.   **.** | N/A |
| Identification of Factors Influencing the Adoption of Health  Information Technology by Nurses Who Are Digitally Lagging:  In-Depth Interview Study | Leeuw et al.,  (2020)  Netherlands | The study explores experiences and needs of nurses lagging in digital competency. | semi-structured interviews | 10 registered  nurses (RN  in Medical  Center | **Characterization of Nurses:**   - Participants are late technology adopters both personally and professionally. - Resistance to IT and technology, inadequate training, and lack of on-the-job support contribute to stress and feelings of incompetence. - Tendency to avoid or postpone Health Information Technology (HIT) use in nursing practice.   **FITT-Framework Dimensions:**  **Individual and Technology Fit:**   - Participants show incompetence with computers, electronic devices, and software. - Lack of enthusiasm for learning about technology. - Resistance to using electronic devices during nursing care. | A limitation of the study is that they cannot generalize the results to other groups or to the general population, as it is a single case study in one Dutch university hospital |
| Healthcare providers' digital competency: a cross-sectional survey in a low-income country setting | Shiferaw et al., (2020)  North-West Ethiopia. | The aim of this study was to assess digital competency of  healthcare | A cross-sectional study design | 167 healthcare providers  In seven public health centers | - Participants are late technology adopters both personally and professionally.  - Resistance to IT and technology, inadequate training, and lack of on-the-job support contribute to stress and feelings of incompetence.  - Participants show incompetence with computers, electronic devices, and software.  - Lack of enthusiasm for learning about technology.  - Participants mentioned several barriers to obtaining basic digital skills and competencies. There was a lack of general digital knowledge, and little or no formal digital training or education. | - **Limitations:** - Study limited by a relatively small sample size. - Future research could explore digital competency across various health professions and competency levels in a larger, more representative sample |
| Acceptance by laypersons and medical  professionals of the personalized eHealth  platform, eHealthMonitor | Griebel et al.,  (2017)  German, Polish, and Greek | The aim of the study is to evaluate the eHealth Monitor by assessing its acceptance factors among both medical professionals and laypersons. | Questionnaires were created on the basis of factors from  literature | *156*  medical professionals and laypersons. | Usability issues, including difficulty in getting familiar with eHealthMonitor, were reported in this study.  Data privacy concerns emerged as a significant barrier to acceptance.  Some laypersons expressed uncertainty about using eHealthMonitor without assistance. | Limiting factors of in this study made it difficult to measure connections and correlations between several items such as the connection between eHealth literacy and the intention to use an eHealth service Although we had a relatively high number of persons who participated in the study (with about 130 medical laypersons and 25 medical professionals in total), there were large differences between the individual groups. |
| Acceptability and uptake of an electronic decision-making tool to support the implementation of IMCI in primary healthcare facilities in KwaZulu-Natal, South Africa | Jensen et al.,  (2020)  Cape  Town, South Africa, | To investigate whether an electronic decision support tool to strengthen IMCI implementation  is acceptable to nurses, clinic managers and caregivers at primary care facilities in  KwaZulu-Natal, South Africa | A mixed-methods  approach | 30 caregivers  In  primary care facilities, i.e. primary healthcare  clinics (PHCs) and community health centres (CHCs); | **eIMCI Uptake:**  Factors influencing lower uptake, computer unavailability, staff allocation, low literacy, training time, and workload concerns.  **Staff Deployment and Computer Literacy:**  Computer confidence strongly related to uptake, suggesting a minimum level of computer literacy is required**.**  . | N/A |
| Nurses’ Perspectives of Person-Centered Spinal Cord Injury Rehabilitation in a Digital Hospital  DOI: 10.1097/rnj.0000000000000201 | Burridge et al., (2020)  Australia | The aim of this article is to report on a study of rehabilitation nurses’ experiences of using eMR and their perspectives on its compatibility with PCC in a multidisciplinary inpatient SCI rehabilitation unit | A mixed-methods  approach | The study consisted of three parts. The first part involved observing interactions between clinicians and patients, and recording them in electronic medical records (n=50). The second part comprised a patient experience questionnaire, in which patients evaluated their experience with deMR as part of their care (n=43). Lastly, focus groups were conducted with clinicians (n=9) to explore their experience with eMR | Nurses face daily challenges with electronic medical records (eMRs) by integrating paper-based activities for efficient and timely care. Despite the push for a paperless environment, nurses realize the value of both electronic and paper systems, considering them as complementary methods instead of opposing ones. This adaptation highlights the significance of speedy documentation processes and a preference for a model that provides immediacy in documentation while acknowledging the practical need for paper-based procedures. | -The findings of the study are limited to the specific context in which it was conducted and cannot be generalized to other settings.  - Although a significant number of participants were included, it is possible that data saturation was not achieved. |
| Cultural impact on the intention to use nursing information systems of nurses in Taiwan and China: Survey and analysis.  doi: 10.2196/18078 | Chang et al.,  (2020)  Taiwan and China | This study examined the effects of cultural and other related factors on nurses’intentions to use nursing information systems. | quantitative ve study | This survey was conducted in two hospitals, one in Taiwan and the other in China. A total of 880 questionnaires were distributed, with 440 in each hospital. | This study investigates the factors that affect nurses' intentions to use information systems for nursing. The results of the study confirmed the importance of performance expectancy and social influence in predicting the usage intentions of nursing information systems. The study also found that operational ease is more important to users in accepting societies like China, indicating a need for tailored strategies. Moreover, information literacy positively affects performance and effort expectancies, indicating that it plays a crucial role in promoting the belief that using nursing information systems improves performance and is not effortful. This ultimately enhances usage intentions. Based on these findings, the study recommends leveraging social influences, acknowledging cultural variations, and prioritizing task simplification for effective system design. | This study used both online and paper questionnaires that relied on self-reported responses, which may raise doubts about the reliability of the data. Furthermore, assuming that all other factors are equal, the difference in culture is expected to be the primary reason why effort expectancy was linked to the use of nursing information systems only in China and not in Taiwan. |
| Understanding the Experience of Geriatric Care Professionals in Using Telemedicine to Care for Older Patients in Response to the COVID-19 Pandemic: Mixed Methods Study | Chen et al.,  (2022)  Canada | This study aims to identify the benefits and challenges geriatric care professionals face when using telemedicine technologies with frail older patients, their caregivers, and their families and how to maximize the benefits of this method of providing care. | a mixed methods study | In this study, 22 participants (73%) were geriatric medicine specialists or geriatricians, 5 (17%) were geriatric psychiatrists, and 3 (10%) were geriatric nurse practitioners. Moreover, 28 (93%) participants completed both the survey and the semi-structured interview. | Complexity: There are challenges associated with the complexity of transitioning from providing in-person care to virtual care.(B)  Design Quality and Packaging:  connectivity issues were often still a barrier for both geriatric care professiona.(B)  Inner Setting:  Readiness for Implementation: a few statements also revealed that those who had previous experience with telemedicine use found the transition to be smooth.(F)  Available Resources: Many care professionals had available resources and support for training on virtual care. A few did not initially have infrastructure available to them.(F) | The limitation of this study, the findings are limited to the experiences of geriatric care professionals in Canada’s Greater Toronto Area. In addition, the experience of nonmedical or nursing allied health professionals, who are also integral members of some geriatric care teams, was not included |
| Digital health technology: factors affecting implementation in nursing homes | Curtis et al.,  (2020)  England | To identify the factors that enable nurses to implement DHT in nursing homes and to co-design a nurse-led stepped process supporting the effective implementation of DHT innovations in nursing homes. | An appreciative inquiry methodology | The manager and the two nurses in each nursing home were interviewed individually, while the resident and their relative were interviewed together. This meant that 20 interviews took place in total; four interviews in each nursing home  (managers, residents and relatives, and nurses) | **Managers:**  Participating nursing managers had various experiences of and attitudes towards DHT – from supporting full engagement to believing that technology would not be helpful.   - Nurses   Participating nurses expressed an interest in DHT and regarded its implementation as the future of healthcare. They suggested that achieving successful DHT implementation would enhance the quality of nursing care, provided that the introduction process, cost, security and management were all carefully considered.   - Nurses acknowledged the possibility of Digital Health Technologies (DHT) to enhance healthcare practices, they emphasized the positive impact of DHT on staff communication, enabling real-time sharing of information, The interest in DHT centered on its ability to streamline access to information, contrasting the time-consuming process of navigating paper-based records. Nurses expected that DHT, through features like easy retrieval of data and simplified care plan amendments, could significantly reduce paperwork duties, enhancing overall efficiency in nursing operations. | Limitations The main limitation of the LAUNCH study was its small sample size, which means that findings cannot be generalized to a wider geographical area and/or to other nursing homes. |
| Acceptability and Potential Effectiveness of eHealth Tools for Training Primary Health Workers from Nigeria at Scale: Mixed Methods, Uncontrolled Before-and-After Study | Hicks et al., (2021)  Nigeria | This study aims to determine the feasibility and acceptability of these digital health tools and their potential effectiveness in improving clinical knowledge, attitudes, and practices related to MNCH care. | A mixed methods design, | Included a total of 126 health facilities in three states in Nigeria (Kano, Ondo, and FCT  face-to-face. semi structured qualitative interviews with 34 participants in 3 states—12 FHWs, 12 facility managers, and 10 policy makers. | **Ease of use eHealth tools is linked to perceived accessibility**:  The relationship between the ease of accessing the device and the level of motivation to use the eHealth tools.  **Training videos on the eHealth tools**:  After downloading to tablet computers, the videos can be accessed offline by frontline health workers and facility managers at no extra cost. The videos have become reliable reference materials due to their convenience for repeated use.  **Perceived usefulness for improving service provision:**  A key driver of acceptability cited by FHWs was the perceived usefulness improving the quality of health care provision.  **External barriers to the use eHealth Tools**:  (1) poor internet connection to log into the **e**Health tools (2) poor electricity supply to charge devices, and (3) workload issues that prevented FHWs from completing pre- and post-tests. | First, an uncontrolled before-and-after comparison lacks the robustness and comparability of a randomized experimental design, which could compare an e-learning intervention to conventional face-to-face training.    Second, although interview questions were developed and refined with key stakeholders and interviews conducted by experienced researchers, interview guides were not pilot tested before use. |
| The management of digital competence sharing in health care: A qualitative study of managers' and professionals' views | Hammarén et al., (2023)  Finland | To describe managers' and professionals' views on the management of digital competence sharing in health care. | A qualitative descriptive study. | Participants were managers (n = 22) and professionals (n = 12) from different health care organizations in Finland. | **Providing resources and opportunities for digital competence sharing:**  The first main category consisted of three categories: providing temporal resources for digital competence sharing, freeing up work resources for digital competence sharing and creating opportunities for digital competence sharing. | N/A |
| Medical and nursing clinician perspectives on the usability of the hospital electronic medical record: A qualitative analysis | Lloyd et al., (2023)  Australia | To explore perspectives of medical and nursing clinicians on EMR usability utilising free-text data collected in a survey. | Qualitative analysis of one free-text optional question | (85 doctors and 27 nurses) from 34 professional bodies representing the health clinician disciplines who worked in the Australian healthcare system. | **Multiple logins and sign-outs**  These issues challenged users and led to a loss of productivity through wasted time and is exemplified in the quotes.  **Searchability and information finding.**  Searchability and difficulty in locating information, a limitation of paper systems was also apparent in the EMR systems used. | - This study was conducted during 2020, with data collection affected by the COVID pandemic that disrupted the health system and added burden to the hospital system in most Australian states. |
| Digital technologies in routine palliative care delivery: an exploratory qualitative study with health care professionals in Germany | May et al., (2022)  German | To explore health care professionals’ (HCPs) perspectives, experiences and preferences towards digital technology use in routine palliative care delivery. | exploratory qualitative study | HCPs (n = 19) purposively selected from a sample of settings that reflect routine palliative care delivery | **Coordination of the work process:**  Participants value the shared electronic documentation to inform and facilitate patient care, enabling more efficient work by reducing the time needed to complete documentation and through preventing mistakes and loss of patient data | - The sample included only participants from one federal state in Germany. Possibly, the recruitment of participants via the working group might have led to the involvement of sites more engaged in palliative care research. Furthermore, selection bias may have occurred through expressions of interest potentially arising from providers with an interest in digitalization |
| Adaptation of Remote Symptom Monitoring Using Electronic Patient-Reported Outcomes for Implementation in Real-World Settings | Rocque et al., (2022) | Despite evidence of clinical benefits, widespread implementation of remote symptom monitoring has been limited. they describe a process of adapting a remote symptom monitoring intervention developed in research setting to a real-world clinical setting at two cancer centers. | formative evaluation, qualitative evaluation with semi structured interviews | Physicians (n = 5), nurses/nurse navigators (n = 5), and administrative leaders (n = 2) | -Training nurses to manage alerts,  -Identification of personnel responsible for managing symptoms. | The limitation of this study is the adaptation process and key adaptations for two centers located in the Southern United States. Other sites may differ in their institutional culture, staffing expectations, implementation readiness, and individual barriers experienced. |
